# Safety and efficacy of skin patches containing loxoprofen sodium in diabetic patients with overt nephropathy

**DOI:** 10.1007/s10157-013-0850-4

**Published:** 2013-08-07

**Authors:** Hisazumi Araki, Shogo Kuwagata, Mariko Soumura, Kosuke Yamahara, Yoshikata Morita, Shinji Kume, Keiji Isshiki, Shin-ichi Araki, Atsunori Kashiwagi, Hiroshi Maegawa, Takashi Uzu

**Affiliations:** Department of Medicine, Shiga University of Medical Science, Otsu, Shiga 520-2192 Japan

**Keywords:** Skin patches, Loxoprofen, Diabetic nephropathy, Type 2 diabetes, Prostaglandin E_2_, GFR, NSAIDs

## Abstract

**Background:**

Because oral nonsteroidal anti-inflammatory drugs (NSAIDs) have adverse effects on kidney function, patients with kidney diseases are administered these drugs as transdermal patches. Little is known about the effects of NSAID patches on renal function. We therefore assessed the effects of topical loxoprofen sodium on kidney function in type 2 diabetic patients with overt nephropathy.

**Methods:**

Twenty patients with type 2 diabetes and overt proteinuria and with knee and/or low back pain were treated with skin patches containing 100 mg loxoprofen on the knee or back for 24 h per day for 5 consecutive days. The degree of pain was assessed using a visual analogue scale (VAS). Blood and 24-h urine samples were obtained at baseline and at the end of the study. Glomerular filtration rate (GFR) was estimated from serum creatinine and cystatin C concentrations.

**Results:**

The 20 patients consisted of 11 males and 9 females, of mean age 61.6 ± 13.9 years. Loxoprofen-containing patches significantly reduced VAS pain without affecting blood pressure, GFR or urinary prostaglandin E_2_ concentration. Serum concentrations of loxoprofen and its active trans-OH metabolite did not correlate with GFR.

**Conclusions:**

Loxoprofen-containing patches do not affect renal function in type 2 diabetic patients with overt nephropathy over a short-term period. Long-term studies are needed to clarify the safety of loxoprofen-containing patches in patients with chronic kidney diseases.

## Introduction

Nonsteroidal anti-inflammatory drugs (NSAIDs) are widely used, with acknowledged efficacy and safety over a wide range of clinical conditions. Despite their many useful therapeutic applications, substantial evidence has shown that NSAIDs can have deleterious effects on kidney function. For example, a nested case-controlled study using the General Practice Research Database from the United Kingdom showed that NSAID users in the general population were at threefold greater risk for a first-ever diagnosis of clinical acute kidney injury (AKI) than non-NSAID users. In addition, history of heart failure, hypertension, and diabetes were associated with a greater risk of AKI in this population [1].

Combination therapy with NSAIDs and renin–angiotensin system (RAS) inhibitors increases the risk of kidney damage [2–4]. Since RAS inhibitors are recommended as first-line antihypertensive agents in patients with diabetes, patients with diabetic nephropathy who take NSAIDs tend to be at greater risk for NSAID-induced kidney damage.

NSAIDs can affect renal function by, for example, inhibiting the synthesis of important renal prostaglandins, especially those involved in solute homeostasis and maintenance of renal blood flow [5–8]. Prostaglandin E_2_ (PGE2) is the most abundant vasodilatory prostaglandin in the human renal vascular bed. NSAIDs decrease PGE2 concentration by inhibiting cyclooxygenase-2 (COX-2). Adverse effects of NSAIDs may be avoided by administering these drugs as transdermal patches. These adhesive patches, which are applied to the skin at the site of pain, slowly release medication through the skin. Although NSAID patches are regarded as safe and are frequently used in patients with chronic kidney disease (CKD), the effects of NSAID patches on renal circulation in these patients have not been investigated. Loxoprofen-containing patches are one of the most widely used adhesive patches in Japan. We therefore analyzed the effects of topically applied loxoprofen sodium on kidney function in patients with diabetic nephropathy.

## Methods

### Study design

This open-label, single-arm, single-dose study was performed at the Shiga University of Medical Science Hospital. In patients with type 2 diabetes and overt proteinuria [urinary albumin–creatinine ratio (ACR) >300 mg/g] who had knee and/or low back pain were recruited. We enrolled 20 patients with type 2 diabetes complicated by diabetic nephropathy stage III to IV. Patients with diabetic nephropathy were classified by the Ministry of Health, Labour and Welfare of Japan [9]. The 20 patients consisted of 11 males and 9 females, ranging in age from 34 to 80 years [median age 61.6 years]. Patients previously treated with NSAIDs or with a history of hypersensitivity to NSAIDs were excluded. We also excluded those who underwent knee or spine surgery, and patients with hematologic disease, liver cirrhosis, heart failure or malignancy.

Adhesive skin patches containing 100 mg loxoprofen (LX-P; Loxonin^®^ tape) were applied to the back or knee of each patient, depending on the site of pain, for 24 h per day for five consecutive days (one patch per day). The degree of pain was assessed using a visual analogue scale (VAS) [10], consisting of a straight 10-cm line, presenting a continuum of pain intensity, with ‘no pain’ at the bottom and ‘pain as bad as it can be’ at the top. Blood pressure was measured by an aneroid sphygmomanometer in the supine position before breakfast. The mean blood pressure values of 2 consecutive days before treatment were used as the baseline and the mean blood pressure value of days 4 and 5 were used as the end-point. Blood and urine samples were obtained under fasting conditions at baseline and at the end of the 5-day study period.

The estimated glomerular filtration rate (eGFRcre) of each patient was calculated using the simplified equation of the Japanese Society of Nephrology, a version of the Modification of Diet in Renal Disease study equation modified for Japanese patients [11]. GFR was also estimated from serum cystatin C concentrations (eGFRcys), as recently recommended by the Japanese Society of Nephrology [12]. HbA1c was measured using high-performance liquid chromatography and expressed as the National Glycohemoglobin Standardization Program (NGSP) equivalent value (%), as recommended by the Japanese Diabetes Society. Serum concentrations of loxoprofen and its active, trans-OH metabolite were measured by liquid chromatography coupled with tandem mass spectrometry (LC/MS/MS) (Sumika Chemical Analysis Service, Ltd., Osaka, Japan). Urinary PGE2 concentrations were measured by a chemiluminescence immunoassay (SRL, Inc., Tokyo, Japan).

The study protocol was approved by the Ethics Committee of Shiga University of Medical Science (approval number: 22-83-1), and all participants provided written informed consent.

### Statistical analysis

Data were analyzed using SPSS version 17.0 (SPSS, Tokyo, Japan). The distribution of variables was analyzed by checking histograms and normal plots of the data, and normality was tested using the Kolmogorov–Smirnov and Shapiro–Wilk tests. Student’s *t* test was used to compare values at different time points. Pearson’s or Spearman’s rank correlation coefficients were calculated to determine the correlations between variables. Values were expressed as mean ± SD, and *P* < 0.05 was considered statistically significant.

## Results

The 20 patients enrolled in this study consisted of 11 males and 9 females, ranging in age from 34 to 80 years (median age 61.6 years). The average height of the patients was 157.6 ± 10.8 cm, the average body weight was 69.8 ± 18.6 kg, and their average HbA1c was 7.2 ± 1.4 %. Their mean eGFRcre and eGFRcys were 24.8 ± 17.7 and 35.0 ± 21.1 mL/min/1.73 m^2^, respectively. Two of the patients applied the LX-P on their knee and 18 applied the patch on their back.

**Fig. 1:**
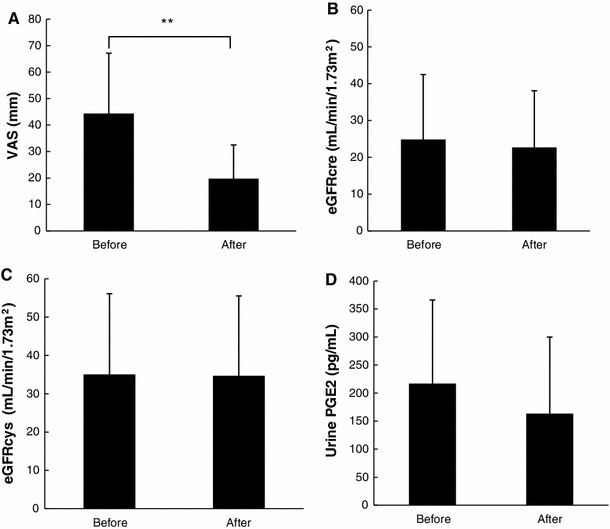
Effects of topically administered LX-Ps on (**a**) pain VAS, (**b**) eGFRcre, (**c**) eGFRcys, and (**d**) urinary PGE2. **P < 0.01

Their mean systolic and diastolic blood pressure measurements at the end of the LX-P treatment were 133.7 ± 21.5 and 73.2 ± 11.7 mmHg, respectively. Systolic and diastolic blood pressure at the end of treatment did not differ significantly from baseline (*P* = 0.211 and *P* = 0.843, respectively). Pain assessed on a 10-point VAS was significantly reduced by LX-Ps (Fig. 1a), whereas renal function, assessed by eGFRcre and eGFRcys, was not affected (Fig. 1b, c). In addition, urinary PGE2 concentrations did not change from baseline to the end of therapy (Fig. 1d). These results indicated that, in patients with type 2 diabetes and overt proteinuria, LX-Ps reduced pain without affecting renal microcirculation.

**Fig. 2:**
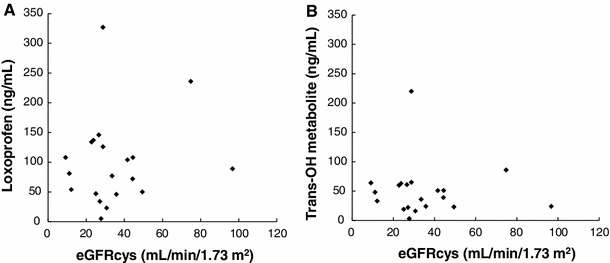
Correlations between eGFRcys and the absorption of loxoprofen sodium. The correlation of eGFRcys and serum concentration of (**a**) loxoprofen sodium (*r* = 0.15, *P* = 0.53) and (**b**) the trans-OH metabolite of loxoprofen sodium (*r* = − 0.073, *P* = 0.76)

The mean ± SD serum concentrations of loxoprofen and its trans-OH metabolite at the end of the 5-day LX-P treatment period were 100.2 ± 75.0 and 50.4 ± 45.2 ng/mL, respectively. These concentrations did not correlate with renal function (Fig. 2a, b).

PGE2 concentrations in fasting urine before and after the administration of LX-Ps did not differ significantly (216.9 ± 149.3 and 163.3 ± 136.9 pg/mL, *P* = 0.23) (Fig. 1d). Moreover, there was no correlation between the concentration of PGE2 and eGFRcys, either before (*r* = −0.16, *P* = 0.51) or after (*r* = −0.14, *P* = 0.55) treatment with LX-Ps (data not shown).

## Discussion

Although the serum concentrations of loxoprofen sodium have been measured following oral administration in patients without renal impairment, these concentrations were not measured in patients with renal impairment. To our knowledge, this study is the first to evaluate serum concentrations of loxoprofen sodium and urinary concentrations of PGE2 following the administration of LX-Ps to patients with diabetic nephropathy.

We found that short-term administration of LX-Ps was effective in treating knee and lower back pain in Japanese patients with diabetic nephropathy, without negatively affecting renal function. All 20 of our patients had overt protein in urine, but their eGFRcre ranged from normal (>60 mL/min/1.73 m^2^) to severe renal impairment (<30 mL/min/1.73 m^2^). Serum concentration of loxoprofen sodium and its trans-OH metabolite following a single oral dose of 60 mg have been reported to be 5.04 ± 0.27 and 0.85 ± 0.02 μg/mL, respectively [13]. We found that both serum concentrations were much lower, 100.2 ± 75.0 and 50.4 ± 45.2 ng/mL, respectively, after the application of transdermal LX-Ps. Moreover, these patches had no effect on PGE2 concentrations. Taken together, these results suggest that topically administered loxoprofen sodium was safer for patients with renal impairment than the orally administered agent.

Loxoprofen sodium and its trans-OH metabolite are both metabolized in and secreted by the liver and kidneys, suggesting that, in patients with renal impairment, their serum concentrations would be higher in patients with AKI than in those with normal renal function. To assess whether serum concentrations of these molecules differed according to renal function, we examined the relationship of each to eGFRcys. However, we did not detect any correlations. These findings indicated that loxoprofen sodium and its active metabolite were not increased in patients with severe renal impairment. This suggests that the absorption of loxoprofen sodium by the systemic circulation is lower when this agent is administered topically than orally, and is therefore not altered by renal function. We predict that the concentration of loxoprofen sodium and its trans-OH metabolite are in equilibrium after five consecutive days, but the details of their pharmacokinetics in patients with renal impairment is still unknown.

We analyzed the correlation between the concentration of loxoprofen sodium or its trans-OH metabolite and urinary PGE2. There was no correlation between the concentrations of loxoprofen sodium and urinary PGE2 (*P* = 0.345), or between the trans-OH metabolite and urinary PGE2 (*P* = 0.370) (data not shown). We postulated that this is because the concentrations of loxoprofen sodium and its trans-OH metabolite were so low and in such a narrow range.

NSAIDs are associated with elevated blood pressure and a higher incidence of hypertension [14–19] because they inhibit the production of prostaglandins. However, we found that topically administered loxoprofen sodium did not significantly affect systolic or diastolic blood pressure, likely because it does not decrease prostaglandins.

In conclusion, in contrast to orally administered loxoprofen sodium, topically administered LX-Ps did not increase serum loxoprofen concentrations or decrease urinary PGE2 concentrations in Japanese patients with type 2 diabetes and renal impairment. Topical LX-Ps had no effect on renal function or on blood pressure in these patients. Although our study was limited by the small number of patients, topical LX-Ps showed good short-term safety and efficacy results in patients with diabetic nephropathy. However, additional large-scale and long-term studies are needed to clarify the safety of LX-Ps in patients with chronic kidney diseases. Furthermore, the effect of LX-Ps in patients on dialysis therapy is currently unclear, suggesting the need for further studies to clarify these effects.
